# Study on Dynamic Strength Characteristics of Sand Solidified by Enzyme-Induced Calcium Carbonate Precipitation (EICP)

**DOI:** 10.3390/ma17204976

**Published:** 2024-10-11

**Authors:** Gang Li, Xueqing Hua, Jia Liu, Yao Zhang, Yu Li

**Affiliations:** 1 Shaanxi Key Laboratory of Safety and Durability of Concrete Structures, Xijing University, Xi’an 710123, China; 2 Guangyuan Natural Gas Co., Ltd., Guangyuan 628000, China

**Keywords:** EICP, solidified sand, dynamic strength, cyclic stress ratio, vibration frequency

## Abstract

Saturated sand foundations are susceptible to liquefaction under dynamic loads. This can result in roadbed subsidence, flotation of underground structures, and other engineering failures. Compared with the traditional foundation reinforcement technology, enzyme-induced calcium carbonate precipitation technology (EICP) is a green environmental protection reinforcement technology. The EICP technology can use enzymes to induce calcium carbonate to cement soil particles and fill soil pores, thus effectively improving soil strength and inhibiting sand liquefaction damage. The study takes EICP-solidified standard sand as the research object and, through the dynamic triaxial test, analyzes the influence of different confining pressure (*σ*_3_) cementation times (*CT*), cyclic stress ratio (*CSR*), dry density (*ρ*_d_), and vibration frequency (*f*) on dynamic strength characteristics. Then, a modified dynamic strength model of EICP-solidified standard sand was established. The results show that, under the same confining pressure, the required vibration number for failure decreases with the increase in dynamic strength, and the dynamic strength increases with the rise in dry density. At the same number of cyclic vibrations, the greater the confining pressure and cementation times, the greater the dynamic strength. When the cementation times are constant, the dynamic strength of EICP-solidified sand decreases with the increase in the vibration number. When cementation times are 6, the dynamic strength of the specimens with *CSR* of 0.35 is 25.9% and 32.4% higher than those with *CSR* of 0.25 and 0.30, respectively. The predicted results show that the model can predict the measured values well, which fully verifies the applicability of the model. The research results can provide a reference for liquefaction prevention in sand foundations.

## 1. Introduction

Sand liquefaction is a physical phenomenon where saturated sand behaves like a liquid under dynamic load and completely loses its bearing capacity. Liquefaction of sand leads to the decrease in soil strength, which may cause disasters such as floating of underground structures, roadbed subsidence, landslides, and bank slope erosion [1,2,3,4], and has a significant impact on production and life [5]. Considering the influence of sand liquefaction on the stability of buildings, a large number of scholars have researched on the reinforcement of sand foundations [6,7,8]. The commonly used foundation treatment methods in engineering include physical, chemical, and biological methods [9]. Among them, enzyme-induced carbonate precipitation (EICP) is a green soil improvement technology, which uses commercial enzymes or enzymes extracted from plants [10,11,12] to catalyze the hydrolysis reaction of urea, generating carbonate ions. Carbonate ions combine with calcium ions to form calcium carbonate precipitates, cementing soil particles, filling soil pores, improving soil strength, and reducing soil permeability and liquefaction potential [13,14,15].

In recent years, many scholars have found that the EICP technique performs well in the study of improving the static properties of soils. Nafisi et al. [16] used EICP techniques to reinforce sands, and the obtained results showed that a higher shear strength and larger dilative strain were observed for MICP (microbe-induced calcium carbonate precipitation)-treated sand compared to EICP-treated sand when treated to the same shear wave velocity. von Wolff et al. [17] concluded that the EICP technology can reduce the porosity of a porous medium by precipitating calcium carbonates. Meng et al. [18] discovered that the compressive strength of sandy soils treated with EICP technology was significantly enhanced. This could be employed for reducing permeability to seal flow paths or for soil stabilization. Liu et al. [19] found that the SWCU (plant-derived crude enzyme from sword bean)-treated sand exhibited the best bio-cementation efficacy and was recommended to enhance soil strength. Wu et al. [20] deemed that the presence of PVA (polyvinyl alcohol) could significantly improve the unconfined compressive strength and surface strength of EICP-solidified aeolian sand as the PVA films enhanced cementation between soil particles and between soil particles and calcium carbonate and because PVA film-wrapped calcium carbonate filled the large-sized pores in the soil. Lin et al. [21] demonstrated that EICP treatments can create a cemented soil zone surrounding the pervious concrete pile and improve the pile capacity and load transfer under compression loading. Some scholars have discovered that EICP technology can not only be used for soil reinforcement but also has more obvious advantages in other engineering applications, such as resistance to liquefaction [22], resistance to wind erosion [23], crack repair [24,25,26], and so on, which enrich the scope of engineering applications of the EICP technology.

Some studies [27,28] have shown that there is a significant difference in the mechanical response of soils under static and dynamic loading. Some researchers have carried out experimental studies on the dynamic properties of EICP-cured soils. Tian et al. [29] studied the improvement effect of different calcium source EICP solutions on the dynamic strength of loess. When the confining pressure is small, the calcium acetate source EICP solution has a better effect on improving the dynamic strength of loess, and when the confining pressure is large, the wood calcium source EICP solution has a better effect on improving the dynamic strength of loess. Yuan et al. [30] noted that the dynamic strain of the earthen after an EICP treatment of the site soil could be reduced with the increase in the dry density, confining pressure, and vibration frequency, but this effect diminishes in turn. Singh et al. [31] discovered that the EICP treatment specimens increased the cyclic resistance of oil-contaminated sand up to 1.5 times by suppressing the excess pore pressure build-up. Tabrizi et al. [32] investigated the strain accumulation pattern of the EICP cemented sands under cyclic traffic loads and evaluated the effects of the initial static loads, confining pressures, the number of cycles, and amplitudes of the cyclic loads. Simatupang et al. [33] studied the effect of saturation during precipitation on the liquefaction resistance of sand treated with EICP through undrained cyclic triaxial tests and confirmed that the amounts of urea and CaCl_2_ needed to obtain a given liquefaction resistance can be significantly reduced by decreasing the degree of saturation during curing.

The EICP technology can significantly improve the strength of soil and reduce its permeability. There is little research on the dynamic characteristics of sand solidified by EICP, and a new dynamic strength model of EICP-solidified standard sand has been proposed in the paper. In this study, the effects of confining pressure, cementation times, cyclic stress ratio, dry density, and vibration frequency on dynamic strength characteristics of EICP-solidified sand were systematically studied through triaxial consolidation undrained shear test under cyclic load. Based on the results of the dynamic triaxial test, a new dynamic strength model of EICP-solidified standard sand was proposed, and its applicability was verified. The research results can provide an important reference for the liquefaction control of sandy soil foundations.

## 2. Materials and Methods

### 2.1. Materials

**Figure 1:**
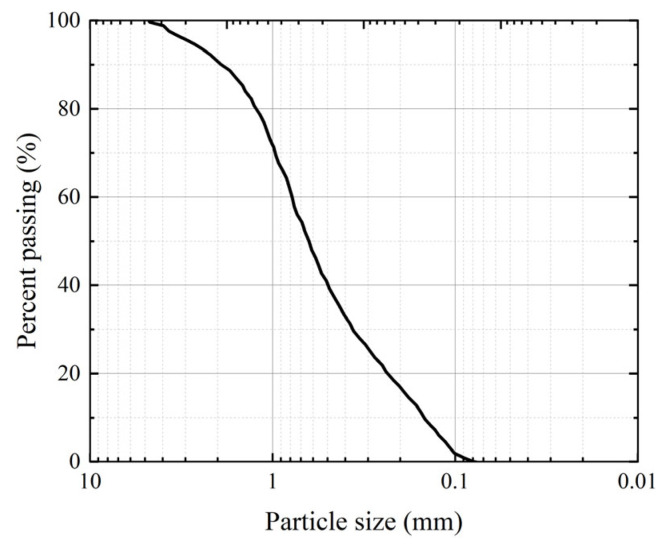
Grading curve of standard sand.

The experimental enzyme was extracted from soybean, which has the advantage of being enzyme-abundant, low cost, and easily obtainable [34]. The soybean was produced in Yilan County Sanxing Ecological Agriculture Co., Ltd. (Harbin, China) and is a non-genetically modified product with round and full particles. The activity of the enzyme was measured using a conductivity meter [35,36]. The steps for enzyme extraction are as follows: the soybeans were dried and ground into powder; after passing through the 0.150 mm sieve, the soybeans were evenly mixed with deionized water and placed on a magnetic stirrer for 30 min; then, they were centrifuged at a speed of 3000 r/min for 30 min before extracting the supernatant, which is the enzyme solution. The experiment used a mixture of urea and anhydrous calcium chloride (China National Pharmaceutical Group Chemical Reagent Co., Ltd., Shanghai, China) as the binder, prepared in a ratio of 1:1 (equal concentration and volume). The standard sand used in the experiment is Chinese ISO standard sand (GB/T 50123-2019) [37] purchased from Xiamen Aisiou Standard Sand Co., Ltd., Xiamen, China. Table 1 shows the physical properties of standard sand. Figure 1 shows the grading curve of standard sand. Based on the curvature coefficient (*C*_c_) and non-uniformity coefficient (*C*_u_), it is determined that the standard sand grading is good.

### 2.2. Sample Preparation

**Figure 2:**
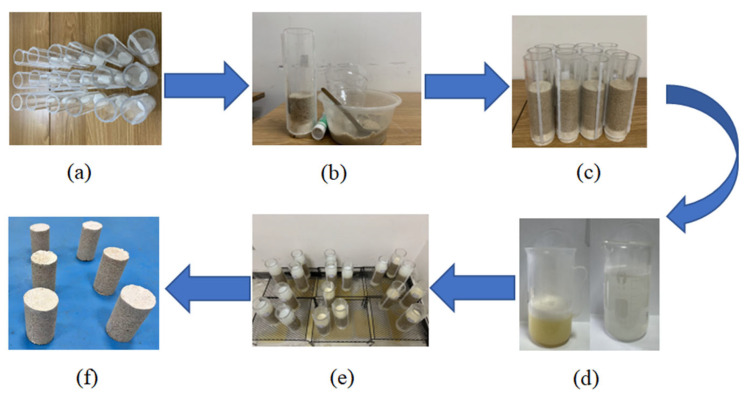
Sample preparation process diagram: (**a**) fixed mold; (**b**) injected standard sand; (**c**) compacted sand column; (**d**) preparation of enzyme solution and cementation solution; (**e**) injection of enzyme solution and cementation solution; and (**f**) sample after curing.

The organic glass tube was selected as the sample mold, with an inner diameter of 50 mm and a height of 200 mm. The sample has a diameter of 50 mm and a height of 100 mm. First, the hot melt gun was used to fix both sides of the mold to prevent leakage during the pouring process. The bottom of the mold was sealed with a rubber plug with a hole, and a layer of filter paper was laid. According to the standard for geotechnical test methods (GB/T 50123-2019) [37], samples were prepared in four layers, with scraping treatment between layers and the compaction of the sand column using a compaction hammer and a layer of filter paper laid on the top of the sample to reduce the impact disturbance of the injected liquid. During the infusion process, firstly, 40 mL of enzyme was poured into the mold, and then, the sample was allowed to stand for 30 min until the enzyme fully penetrated the pores of the sandy soil. After sufficient infiltration, 40 mL of the adhesive solution was injected, and it was allowed to stand for 24 h. The mold was removed, and it was air-dried at room temperature. Figure 2 shows the sample preparation process.

### 2.3. Methods

According to the standard method of geotechnical testing (GB/T 50123-2019) [37], triaxial consolidated undrained shear tests were conducted on standard sand samples. The experiment used the DYNTTS type soil dynamic triaxial testing machine (manufactured by Earth Products China Limited, Hong Kong, China) to load in a stress-controlled manner with a sinusoidal wave load applied axially for 10,000 cycles. The test was stopped when the pore water pressure reached the effective confining pressure. To analyze the dynamic characteristics of EICP-solidified sand under cyclic loading, the influence factors such as confining pressure (*σ*_3_), cementation times (*CT*), cyclic stress ratio (*CSR*), dry density (*ρ*_d_), and vibration frequency (*f*) were considered during the test process. The test plan is shown in Table 2. The specific steps are as follows: the sample is first placed in a vacuum saturation cylinder to saturate for more than 10 h and then taken out and put in a pressure chamber for stepwise back pressure saturation. When the pore water pressure coefficient (*B*) exceeds 0.99, the sample reaches a saturation state. After the sample is saturated, apply effective confining pressure to the sample for consolidation for more than 60 min, and complete the consolidation after there is no change in the drainage volume. A total of 108 sets of experiments were conducted, with each set of samples set up for repeated testing.

## 3. Results

### 3.1. Analysis of the Influence of Confining Pressure on Dynamic Strength

**Figure 3:**
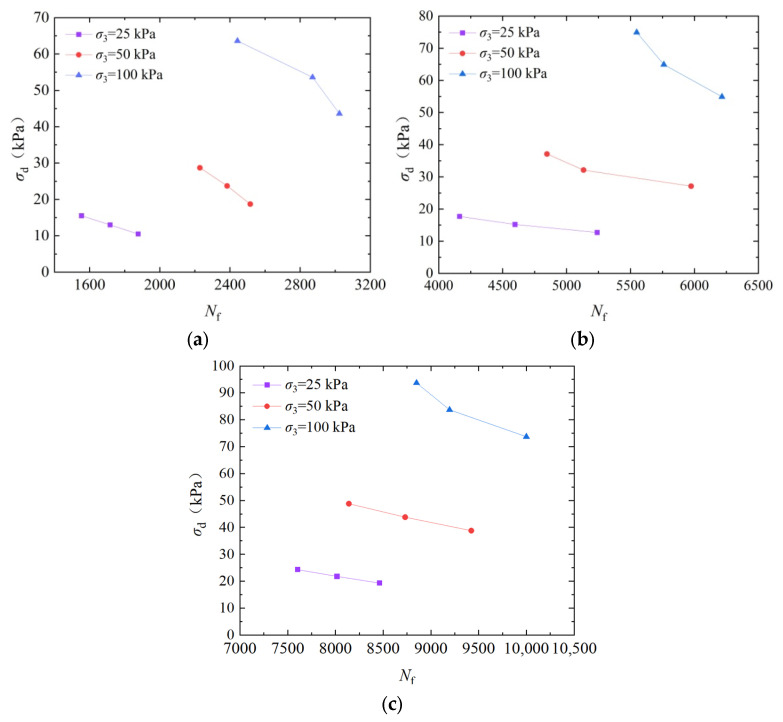
Dynamic strength curves of EICP-solidified standard sand under different confining pressures: (**a**) *CT* = 2; (**b**) *CT* = 4; and (**c**) *CT* = 6.

To analyze the influence of confining pressure on the dynamic strength, Figure 3 shows the dynamic strength curves of EICP-cured standard sand for dry density at 1.70 g/cm^3^ and vibration frequency at 3 Hz. In Figure 3, under the same confining pressure, the required number of vibrations for specimen failure decreases with the increase in dynamic strength [38]. When the cyclic vibration frequency is the same, the greater the confining pressure and cementation times, the greater the dynamic strength. When the confining pressure is 100 kPa, compared with the samples treated with confining pressures of 25 kPa and 50 kPa, the dynamic strength of the samples after cementation times are 2 increased by 274.8% and 95.6%, respectively; when cementation times are 4, the dynamic strength of the sample increased by 338.1% and 163.4%, respectively; when cementation times are 6, the dynamic strength of the sample increased by 412.8% and 205.6%, respectively. The main reason for the above phenomenon is that the calcite crystals generated by EICP cementation are wrapped around the surface of the sand particles or filled in soil particles, and these generated structures enhance the strength of the connection between the soil particles so that the cohesion of the soil and the angle of internal friction is improved. The increase in confining pressure results in tighter stress transfer between sand particles, increased friction, and improved shear strength [39].

### 3.2. Analysis of the Influence of Cementation Times on Dynamic Strength

**Figure 4:**
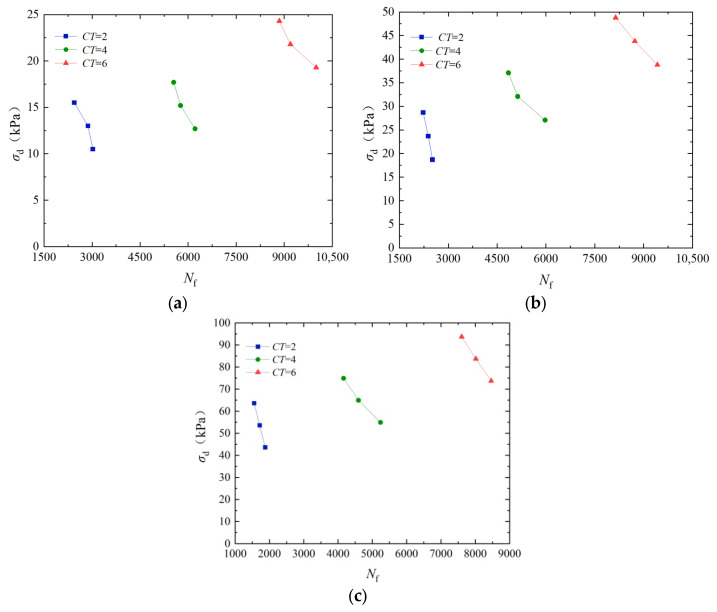
Dynamic strength curves of EICP-solidified standard sand under different cementation times: (**a**) *σ*_3_ = 25 kPa; (**b**) *σ*_3_ = 50 kPa; and (**c**) *σ*_3_ = 100 kPa.

To analyze the influence of cementation times on the dynamic strength, Figure 4 shows the dynamic strength curves of EICP-cured standard sand for a dry density of 1.70 g/cm^3^ and vibration frequency of 3 Hz. In Figure 4, the dynamic intensity decreases with the increase in vibration number; under the same vibration number, the higher the degree of microbial reinforcement, greater the dynamic strength. When confining pressure is 50 kPa, the dynamic strength of the specimen with the cementation times of 6 increased by 154.3% and 108.4% compared to that of the specimen whose cementation times are 2 and 4, respectively. When confining pressure is 100 kPa, the dynamic strength of the specimen with the cementation times of 6 increased by 175.7% and 127.1% compared to that of the specimen whose cementation times are 2 and 4, respectively. The main reason for the above phenomenon is that, with the increase in the cementation times, CaCO_3_ crystals produced by EICP mineralization gradually increase, and the contact between particles gradually changes from point contact to surface contact. The generated structure enhances the connection strength between soil particles, makes loose soil samples consolidate into a whole structure, and improves the cohesion and internal friction angle of soil. At the same time, the generated CaCO_3_ can fill the gap between particles and reduce the porosity of samples, thus increasing the dynamic strength of sand [40].

### 3.3. Analysis of the Influence of Cyclic Stress Ratio on Dynamic Strength

**Figure 5:**
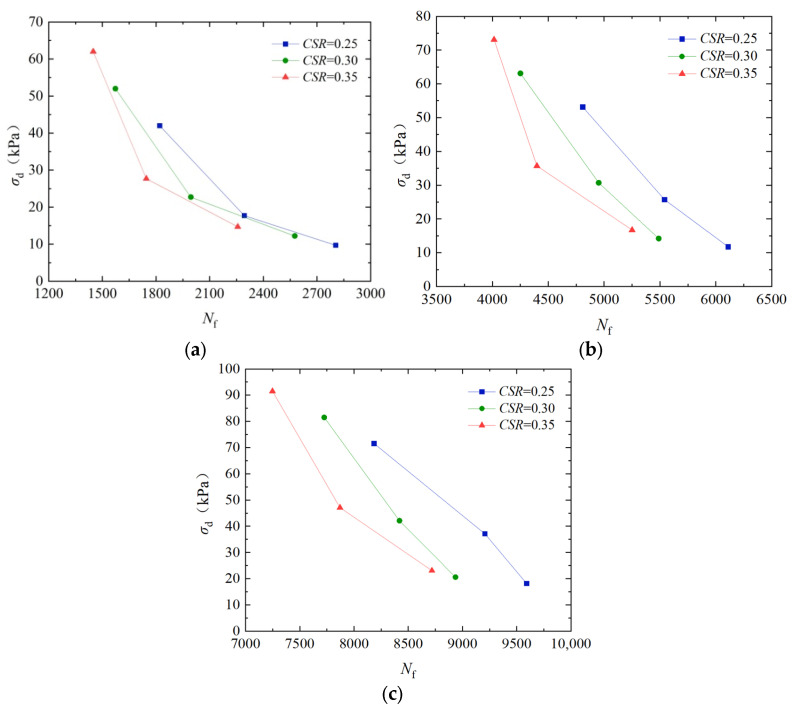
Dynamic strength curves of EICP-solidified standard sand under different cyclic stress ratios: (**a**) *CT* = 2; (**b**) *CT* = 4; and (**c**) *CT* = 6.

To analyze the influence of *CSR* on the dynamic strength, Figure 5 shows the dynamic strength curves of EICP solidification standard sand at a dry density of 1.70 g/cm^3^ and a vibration frequency of 1 Hz. As shown in Figure 5, when the cementation times are constant, the dynamic strength of EICP-solidified sand decreases with the increase in vibration cycles. When cementation times are 6, the dynamic strength of the specimen with a *CSR* of 0.35 increased by 25.9% and 32.4% compared to the specimens with *CSRs* of 0.25 and 0.30, respectively. As the number of vibrations remains constant, the dynamic strength of solidified sand decreases with an increase in the *CSR*. When the dynamic strength is constant, the number of vibration failures of solidified sand grows with an increase in the *CSR*. The main reason for the above phenomenon is that the EICP process generates calcite cement, which increases the bonding force between soil particles, enhances the shear strength of the soil, and increases the failure frequency of the sample; as the *CSR* increases, the relative sliding and displacement between particles increase, and the deformation and stress acting on the specimen increase, thereby enhancing the frictional resistance and cohesion between particles and increasing the dynamic strength of sand.

### 3.4. Analysis of the Influence of Dry Density on Dynamic Strength

**Figure 6:**
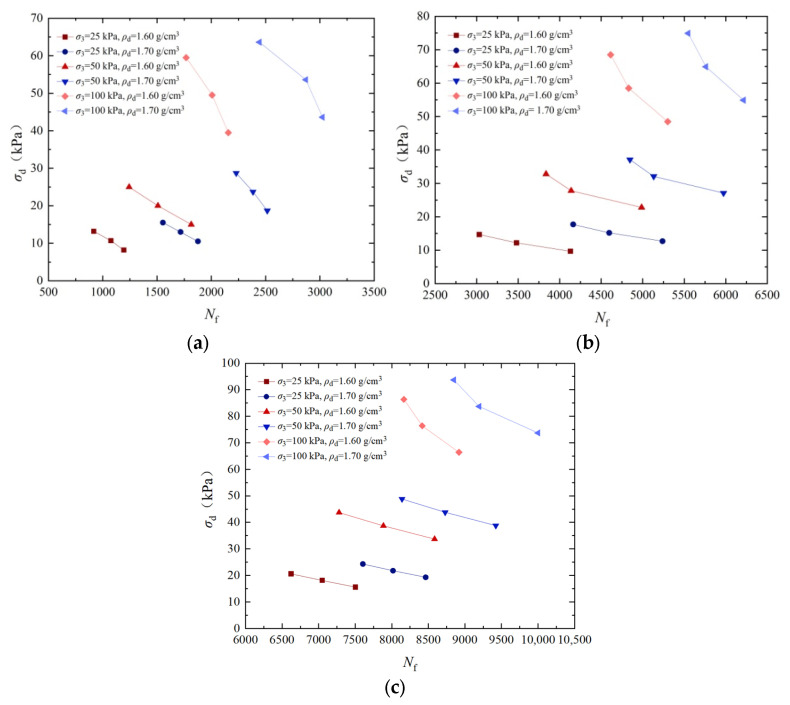
Dynamic strength curves of EICP-solidified standard sand under different dry densities: (**a**) *CT* = 2; (**b**) *CT* = 4; and (**c**) *CT* = 6.

To analyze the influence of dry densities on the dynamic strength, Figure 6 shows the dynamic strength curves of EICP-solidified standard sand at a certain confining pressure and when the vibration frequency is 3 Hz. It can be seen in this figure that, under certain confining pressures and cementation times, the dynamic strength of the sample increases with the increase in dry density. With the increase in cementation times, the influence of dry density on dynamic strength tends to weaken. The dynamic strength of dense sand under 25 kPa, 50 kPa, and 100 kPa confining pressures is 57.6%, 50.4%, and 39.5% higher than those of loose sand after 6 times of cementation treatments. The main reason for the above phenomenon is that the higher dry density leads to the reduction in pore structure and the relatively small damping effect, which reduces the deformation ability of sand under dynamic loading. At the same time, the higher dry density usually is related to the closer particle arrangement and smaller pore space. This structure will make the sand have better contact between particles and closer connection between particles, and the stronger the interaction within the sand, the higher is the corresponding increase in the dynamic strength [41].

### 3.5. Analysis of the Influence of Vibration Frequency on Dynamic Strength

**Figure 7:**
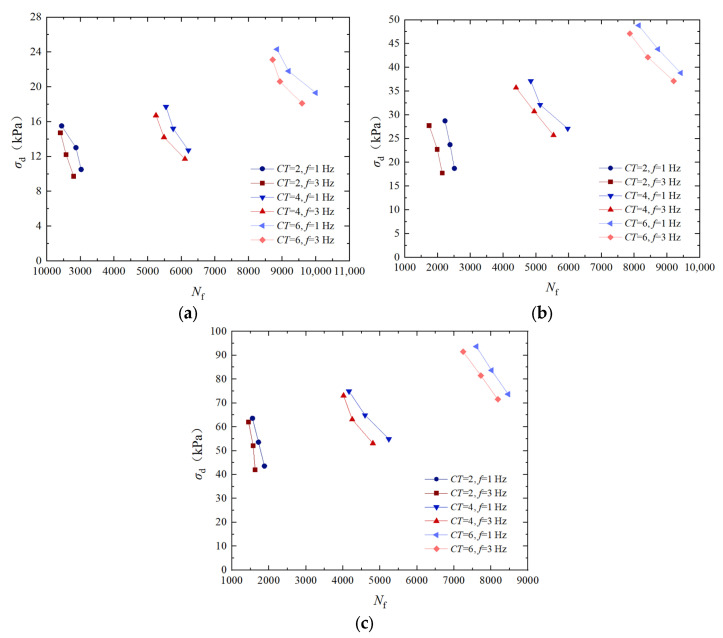
Dynamic strength curves of EICP-solidified standard sand under different vibration frequencies: (**a**) *σ*_3_ = 25 kPa; (**b**) *σ*_3_ = 50 kPa; and (**c**) *σ*_3_ = 100 kPa.

To analyze the influence of vibration frequency on the dynamic strength, Figure 7 shows the dynamic strength curve of EICP solidification standard sand at certain cementation times and at a dry density of 1.70 g/cm^3^. The dynamic strength and the anti-liquefaction ability of EICP-solidified specimens can be enhanced with the increase of confining pressure, cementation times, dry density, and vibration frequency, but decrease with the increase in the cyclic stress ratio. Under certain confining pressures and cementation times, the dynamic strength of the specimen decreases with the increase in vibration frequency. At a certain vibration frequency, the dynamic strength increases with the vibration frequencies and cementation times. When confining pressure is 50 kPa, the dynamic strength of the standard sand with a vibration frequency of 3 Hz is increased by 23.7%, 26.3%, and 32.8% at 2, 4, and 6 times of EICP cementation compared to that at a vibration frequency of 1 Hz, respectively. The main reason for the above phenomenon is that, in a lower frequency range, sand particles have enough time to rearrange under vibration, thereby enhancing the contact and connection between particles. As the frequency increases, the vibration period becomes shorter, and the movement and rearrangement time of soil particles completed within this period decreases. Therefore, the contact enhancement effect between sand particles is relatively weakened, leading to a gradual decrease in the increase in dynamic strength.

### 3.6. Analysis of the EICP Solidification Mechanism

**Figure 8:**
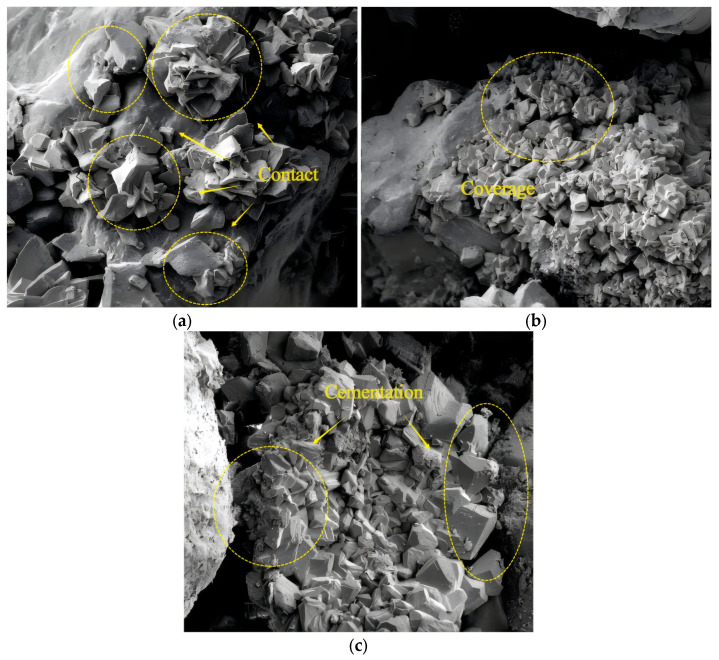
SEM image: (**a**) *CT* = 2; (**b**) *CT* = 4; and (**c**) *CT* = 6.

Figure 8 shows the SEM images of EICP-solidified sand at different cementation times when the dry density is 1.6 g/cm^3^. As shown in Figure 8a, the specimen forms angular or sheet-like calcium carbonate precipitates after EICP treatment, which adhere to the surface of the sand particles [42]. The sand particles are mainly in point contact, and the pores between the sand particles are partially filled with calcium carbonate. Compared to Figure 8b, as the cementation times increase, the precipitation of calcium carbonate on the surface of sand particles significantly increases, and the sand particles are wrapped more tightly. In Figure 8c, the contact mode between particles has changed from point contact to surface contact, enhancing the connection strength between soil particles, and the originally loose sand particles are bonded into a whole [43]. This change significantly increased the cohesion and internal friction angle of the soil, verifying that with the increase in cementation times, the dynamic strength and anti-liquefaction ability also correspondingly improved.

## 4. Dynamic Strength Model of EICP-Solidified Standard Sand

### 4.1. Modeling

According to the dynamic strength curve of EICP-solidified standard sand and its development law, it is found that the dynamic strength variation law under different working conditions can be better expressed by the following formula:

(1) $$\sigma_{d}=\frac{m}{N_{f}}-n$$

where *σ*_d_ refers to the dynamic load (kPa); *N*_f_ represents the cyclic failure frequency (times); *m*, *n* are the empirical parameters (dimensionless).

The Origin 2021 (version 9.8) software and regression analysis methods were used to fit the experimental data, taking into account the influence of *R*^2^ values. Fitted by the above function expression, the fitting results of empirical parameters *m*, *n*, and *R*^2^ of the dynamic strength curve under various working conditions are as Table 3.

According to the analysis results of the dynamic strength curve of EICP-solidified standard sand, there is an inversely proportional relationship between dynamic strength and failure vibration number. According to a previous optimization study of empirical formula parameters, the parameter n has a small change range, so it can be simplified as a test constant, while parameter m can be regarded as a function related to test variables, so it can be expressed as a function in the following form:

(2) $$m=F\left(\sigma_{3},\rho_{d},CT\right)$$

where *m* refers to the empirical parameter (dimensionless); *σ*_3_ represents the confining pressure (kPa); *ρ*_d_ is the dry density of sand (g/cm^3^); and *CT* represents the cementation times (dimensionless).

According to the dynamic strength curve and the observation of the variation law between *m* value and confining pressure, the following functional expressions are selected for fitting:

(3) $$\sigma_{d}=\frac{a_{1}\sigma_{3}\rho_{d}\left[a_{2}{\left(CT\right)}^{2}+a_{3}\left(CT\right)+a_{4}\right]}{\sigma_{r}\times N_{f}}-n$$

where *σ*_d_ is the dynamic load; *σ*_3_ refers to the confining pressure (kPa); *σ*_r_ represents the equilibrium dimensional reference stress (kPa), 1 kPa; *ρ*_d_ is the dry density of sand (dimensionless); *CT* represents the cementation times (dimensionless); *N*_f_ refers to the cyclic failure frequency (dimensionless); *a*_1_, *a*_2_, *a*_3_, *a*_4_ represents the fitting coefficients (dimensionless); *n* is the empirical parameter (dimensionless).

### 4.2. Parameter Fitting

Based on the above-mentioned fitting formulas, the study used the least square method to calculate the optimized dynamic strength empirical parameters *m*, *n* and *R*^2^ of each sample under different confining pressures, cementation times, dry density and vibration frequency. Table 4 summarizes the model parameter values.

Based on Formula (3) and combined with different experimental conditions, the fitting coefficients *a*_1_, *a*_2_, *a*_3_, and *a*_4_ are obtained to be 10^4^, 3.2, 1.8, and 0.7, respectively. The empirical parameter *n* is 25. The dynamic strength model of EICP-solidified standard sand is as follows:

(4) $$\sigma_{d}=\frac{\sigma_{3}\rho_{d}\left[3.2{\left(CT\right)}^{2}+1.8\left(CT\right)+0.7\right]\times 10^{4}}{\sigma_{r}\times N_{f}}-25$$

where *σ*_d_ is the dynamic load (kPa); *σ*_3_ refers to the confining pressure (kPa); *σ*_r_ represents the equilibrium dimensional reference stress (kPa); *ρ*_d_ refers to the dry density of sand (dimensionless); *CT* is the cementation times (dimensionless); and *N*_f_ refers to the cyclic failure frequency (dimensionless).

### 4.3. Model Validation

**Figure 9:**
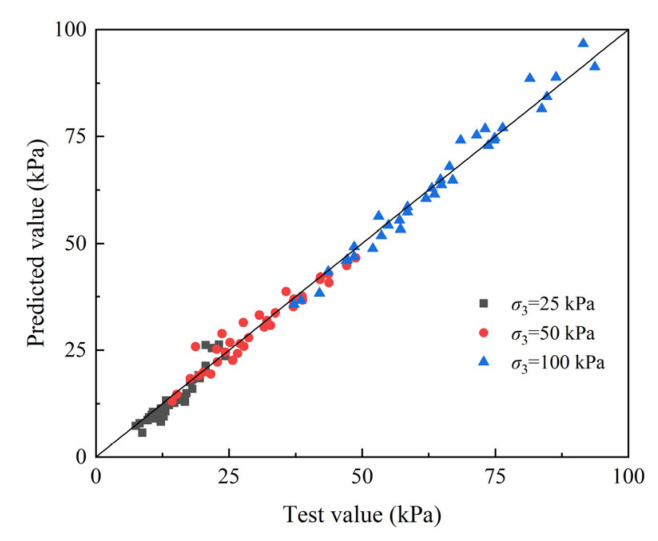
Comparison of dynamic strength between test results and predicted results.

To further verify the applicability of the dynamic strength model in the study, the predicted dynamic strength of EICP-solidified sand was calculated by substituting the values of *σ*_3_, *ρ*_d_, *CT*, *σ*_r_, and *N*_f_ under different experimental conditions into Formula (4). The comparison between the test values and predicted values of the dynamic strength of EICP-solidified sand is shown in Figure 9. As shown in this figure, the dynamic strength is evenly distributed on both sides of the bisector, indicating that the model calculation results are in good agreement with the experimental results. Therefore, the dynamic strength model proposed in this study is the better way to simulate the development law of the dynamic strength curve of EICP-solidified standard sand under different conditions. It is important to highlight that the reasons for individual data deviating significantly from the mean are as follows: firstly, this may be due to the specimen strength deviation caused by the inhomogeneity of EICP strengthening; secondly, the deviation of test data may be caused by the influence of dynamic triaxial specimen preparation and cyclic loading test.

## 5. Conclusions

Based on the dynamic triaxial tests of EICP-solidified standard sand under cyclic loading, the effects of confining pressure, cementation times, cyclic stress ratio, dry density, and vibration frequency on dynamic strength characteristics were systematically studied. The results of dynamic triaxial tests were used to establish a dynamic strength model of EICP-solidified sand, which takes into account the influence of various factors. The main conclusions are as follows:

1. Under the condition of the same confining pressure, the failure cyclic number of specimens decreases with the increase in dynamic strength. Under the same cyclic vibration number, the greater the confining pressure and cementation times, the greater the dynamic strength. When confining pressure is 100 kPa, the dynamic strength of the specimens treated with confining pressures of 25 kPa and 50 kPa increased by 274.8% and 95.6%, respectively; when cementation times are 4, the dynamic strength of the samples increased by 338.1% and 163.4%. The dynamic strength of the specimens after 6 cementation treatments increased by 412.8% and 205.6%.
2. The dynamic intensity decreases with the increase in vibration number. Under the same vibration number, the higher the cementation times, the greater the dynamic strength. When confining pressure is 50 kPa, the dynamic strength of the specimen with cementation times of 6 increased by 154.3% and 108.4% compared to that of the specimen after cementation times of 2 and 4, respectively. When confining pressure is 100 kPa, the dynamic strength of the specimen with cementation times of 6 increased by 175.7% and 127.1% compared to that of the specimen after cementation times of 3 and 4, respectively.
3. When the cementation times are constant, the dynamic strength of EICP-solidified sand decreases with the increase in vibration cycles. When cementation times are 6, the dynamic strength of the specimen with a *CSR* of 0.35 increased by 25.9% and 32.4% compared to the specimens with a *CSR* of 0.25 and 0.30, respectively. As the number of vibrations remains constant, the dynamic strength of solidified sand decreases with an increase in the *CSR*. Conversely, when the dynamic strength is constant, the number of vibration failures of solidified sand decreases with an increase in the *CSR*.
4. Under certain confining pressure and cementation times, the dynamic strength of the specimen increases with the increase in dry density. With the increase in cementation times, the influence of dry density on dynamic strength tends to weaken. The dynamic strength of dense sand under confining pressures of 25 kPa, 50 kPa, and 100 kPa is 57.6%, 50.4%, and 39.5% higher than that of loose sand, respectively, after six reinforcing treatments.
5. Under certain confining pressure and cementation times, the dynamic strength decreases with the increase in vibration number. At a certain vibration frequency, the dynamic strength increases with the vibration frequency and the number of cementation times. When confining pressure is 50 kPa, the dynamic strength of standard sand with a vibration frequency of 3 Hz increased by 23.7%, 26.3%, and 32.8%, respectively, under EICP cementation times of 2, 4, and 6 times compared to a vibration frequency of 1 Hz.
6. The experimental results are in good agreement with the calculated results of the model, which verifies the reliability of the dynamic strength model of EICP-solidified standard sand and shows that the model is suitable for describing the dynamic strength curve prediction of EICP-solidified standard sand.

## Figures and Tables

**Table 1:** Basic physical properties of standard sand.

Specific Gravity *G*_s_	Maximum Dry Density *ρ*_d max_ (g/cm^3^)	Minimum Dry Density *ρ*_d min_ (g/cm^3^)	Effective Diameter *d*_10_ (mm)	Median Diameter *d*_30_ (mm)	Constrained Diameter *d*_60_ (mm)	Coefficient Uniformity *C*_u_	Curvature Coefficient *C*_c_
2.65	1.73	1.46	0.14	0.50	0.90	6.43	1.98

**Table 2:** Main control variable during dynamic triaxial consolidated undrained test.

Confining Pressure *σ*_3_ (kPa)	Cementation Number *CT*	Cyclic Stress Ratio *CSR*	Dry Density *ρ*_d_ (g/cm^3^)	Vibration Frequency *f* (Hz)
25	2	0.25, 0.30, 0.35	1.60, 1.70	1, 3
	4	0.25, 0.30, 0.35	1.60, 1.70	1, 3
	6	0.25, 0.30, 0.35	1.60, 1.70	1, 3
50	2	0.25, 0.30, 0.35	1.60, 1.70	1, 3
	4	0.25, 0.30, 0.35	1.60, 1.70	1, 3
	6	0.25, 0.30, 0.35	1.60, 1.70	1, 3
100	2	0.25, 0.30, 0.35	1.60, 1.70	1,3
	4	0.25, 0.30, 0.35	1.60, 1.70	1, 3
	6	0.25, 0.30, 0.35	1.60, 1.70	1, 3

**Table 3:** Fitting values of empirical parameters of dynamic strength.

Number	Confining Pressure *σ*_3_ (kPa)	Empirical Parameter *m*	Empirical Parameter *n*	Coefficient of Determination *R*^2^
1	25	140,592	122	0.889
2	25	77,218	25	0.954
3	25	249,506	109	0.874
4	25	179,970	52	0.993
5	25	36,199	9	0.791
6	25	39,268	6	0.791
7	25	90,082	24	0.827
8	25	194,139	58	0.987
9	25	38,614	11	0.874
10	25	47,346	13	0.932
11	25	85,602	21	0.995
12	25	57,718	8	0.816
13	50	252,300	22	0.953
14	50	228,015	7	0.892
15	50	469,562	45	0.931
16	50	404,513	23	0.996
17	50	130,533	7	0.991
18	50	159,114	9	0.915
19	50	212,804	13	0.995
20	50	243,341	14	0.894
21	50	122,410	15	0.922
22	50	172,417	23	0.939
23	50	177,628	18	0.906
24	50	250,404	28	0.938
25	100	930,523	64	0.994
26	100	1,129,342	84	0.837
27	100	1,262,393	82	0.996
28	100	1,500,164	104	0.928
29	100	427,610	19	0.975
30	100	478,155	22	0.958
31	100	540,521	22	0.933
32	100	597,832	25	0.947
33	100	368,346	27	0.883
34	100	470,515	37	0.943
35	100	446,292	29	0.866
36	100	369,642	18	0.923

**Table 4:** Fitted values of empirical parameters of dynamic strength after optimization.

Number	Confining Pressure *σ*_3_ (kPa)	Empirical Parameter *m*	Empirical Parameter *n*	Coefficient of Determination *R*^2^
1	25	138,574	25	0.989
2	25	175,670	25	0.954
3	25	265,821	25	0.974
4	25	295,442	25	0.993
5	25	173,442	25	0.894
6	25	204,436	25	0.931
7	25	276,629	25	0.927
8	25	340,281	25	0.987
9	25	232,964	25	0.974
10	25	255,299	25	0.932
11	25	341,083	25	0.995
12	25	363,974	25	0.916
13	50	564,291	25	0.953
14	50	617,101	25	0.992
15	50	791,735	25	0.931
16	50	851,314	25	0.996
17	50	566,115	25	0.991
18	50	625,537	25	0.915
19	50	736,849	25	0.995
20	50	798,485	25	0.894
21	50	609,106	25	0.922
22	50	646,270	25	0.939
23	50	748,541	25	0.906
24	50	786,817	25	0.938
25	100	1,309,075	25	0.994
26	100	1,382,208	25	0.837
27	100	1,551,028	25	0.996
28	100	1,632,010	25	0.928
29	100	1,191,955	25	0.975
30	100	1,247,250	25	0.958
31	100	1,368,649	25	0.933
32	100	1,427,530	25	0.947
33	100	1,134,232	25	0.883
34	100	1,171,359	25	0.943
35	100	1,273,510	25	0.866
36	100	1,319,674	25	0.923

## Data Availability

Data are contained within the article.
